# Immunogenicity and safety of the booster BNT162b2 vaccine in patients with axial spondyloarthritis treated with biological disease-modifying drugs

**DOI:** 10.3389/fimmu.2022.1010808

**Published:** 2022-09-23

**Authors:** Jitka Smetanova, Tomas Milota, Michal Rataj, Jana Hurnakova, Hana Zelena, Anna Sediva, Rudolf Horvath

**Affiliations:** ^1^ Department of Immunology, Second Faculty of Medicine Charles University and Motol University Hospital, Prague, Czechia; ^2^ Department of Paediatric and Adult Rheumatology, Motol University Hospital, Prague, Czechia; ^3^ Department of Virology, Public Health Institute, Ostrava, Czechia

**Keywords:** axial spondyloarthritis, COVID-19, vaccination, immunogenicity, safety

## Abstract

**Background:**

Vaccination confers relatively short-term protection against severe acute respiratory syndrome coronavirus-2 (SARS-CoV-2), indicating the need for booster doses. Immunocompromised individuals, including those with immune-mediated inflammatory diseases (IMIDs), may have pronounced immune response waning. Vaccine-boosted humoral and T-cell responses minimize poor coronavirus disease 19 (COVID-19) outcome without increasing adverse events (AE). There is limited evidence of third-dose vaccination in axial spondyloarthritis (AxSpA) patients. We investigated immune-response persistence after primary vaccination and immunogenicity and safety after the BNT162b2 booster vaccination.

**Methods:**

This prospective observational study enrolled an AxSpA cohort treated with interleukin-17 (IL-17) and tumor necrosis factor-alpha (TNFα) inhibitors. Serum SARS-CoV-2-specific and virus-neutralizing antibodies for humoral response and flow cytometric detection of intracellular cytokines following SARS-CoV-2-specific peptide-based stimulation for T-cell immune responses were assessed, and safety was evaluated *via* a clinical questionnaire.

**Results:**

Fifteen male AxSpA patients treated with TNFα (73·3%) or IL-17 (26·7%) inhibitors were enrolled and had humoral response persistence at 6 months: 905·6 ( ± 186·1 SD) and 409·1 ( ± 335·7) U/mL. Specific antibody concentrations further increased after booster vaccination to 989·7 ( ± 12·62) and 1000 U/mL and T-cell responders from 53·3% to 80%, with no differences between AxSpA (including “vaccination only” and “hybrid immunity” subgroups) and healthy control (HC) cohorts. No severe AE occurred; the AE spectrum was comparable to that of the general population.

**Conclusion:**

Immune-response persistence after primary vaccination and immunogenicity after booster vaccination were unaffected by anti-IL17 or anti-TNFα therapy with similar AE as in the general population.

## Introduction

The coronavirus disease 19 (COVID-19), caused by the severe acute respiratory syndrome coronavirus 2 (SARS-CoV-2), has become a global concern that severely affects patients with risk factors, such as diabetes mellitus and cardiovascular or respiratory tract diseases (1). Patients with immune-mediated inflammatory diseases (IMIDs), despite immune system dysregulation, do not have an increased risk of severe COVID-19 compared to the general population (2). At present, there is no cure for COVID-19, despite their short-term efficacy (3), fail in the long term because of their significant socioeconomic impact (4). Therefore, vaccination remains an essential strategy to control disease spread and alleviate disease severity (5, 6). Currently, two different mRNA (Comirnaty, Pfizer/BioNTech and Spikevax, Moderna), two viral vector (Vaxzevria, AstraZeneca and COVID-19 Vaccine Janssen, Janssen/Johnson & Johnson), and one recombinant subunit vaccine (Novaxovid, Novavax) have been authorized by the European Medicines Agency (EMA) for the prevention of COVID-19, and have shown high levels of immunogenicity and efficacy in the general population that have been confirmed by real-world evidence (7, 8). Furthermore, the booster dose of vaccines has conferred increased protection against the symptomatic course of COVID-19 and decreased the risk of hospitalization and death (9). Although glucocorticoids, mycophenolate mofetil, rituximab, and abatacept attenuated the post-vaccination immune response, there were no differences in immunogenicity in patients who were receiving interleukin-17 (IL-17) or tumor necrosis factor alpha (TNFα) inhibitors (10–12).

Nonetheless, despite the continuously expanding evidence on the waning of specific immunity and response to booster vaccination, there exists a knowledge gap with regard to the effect in immune-mediated inflammatory diseases (IMIDs), including axial spondyloarthritis (AxSpA). Therefore, we assessed the kinetics of the cellular and humoral response 6 months after the second dose and investigated the effect and safety of a third booster dose of the BNT162b vaccine in patients with AxSpA who were treated with biological drugs (bDMARDs), such as adalimumab (ADA) or secukinumab (SEC). ADA and SEC are fully human monoclonal antibodies targeting TNFα and IL-17, respectively, indicated in AxSpA patients with high disease activity after the failure or intolerance of nonsteroidal anti-inflammatory drugs (NSAIDs) (13).

## Methods

### Study design

This prospective observational study was conducted according to the Strengthening the Reporting of Observational Studies in Epidemiology (STROBE) recommendations (completed STROBE checklist) (14), and focused on the immunogenicity after the third booster dose of the BNT162b2 vaccine (primary endpoint); safety of the booster dose and persistence of immune response after 6 months since the primary vaccination schedule in patients with AxSpA (secondary endpoints). The follow-up period was divided into three study-related visits.

**- Visit 1 (screening):** Patient recruitment, inclusion criteria assessment (see *Study population*), and clinical data collection.
**- Visit 2 (baseline, Day 0):** Baseline disease activity assessment and blood sample collection (for humoral and T-cell immune response assessment) before vaccine administration, and vaccination was performed, according to the EULAR recommendations (15), by a dispensary rheumatologist at the national referral center for immunization.
**- Visit 3 (follow-up, Day 30 ± 7):** Clinical and safety data collection and blood sample collection (for humoral and T-cell immune response assessment).

**Figure 1:**
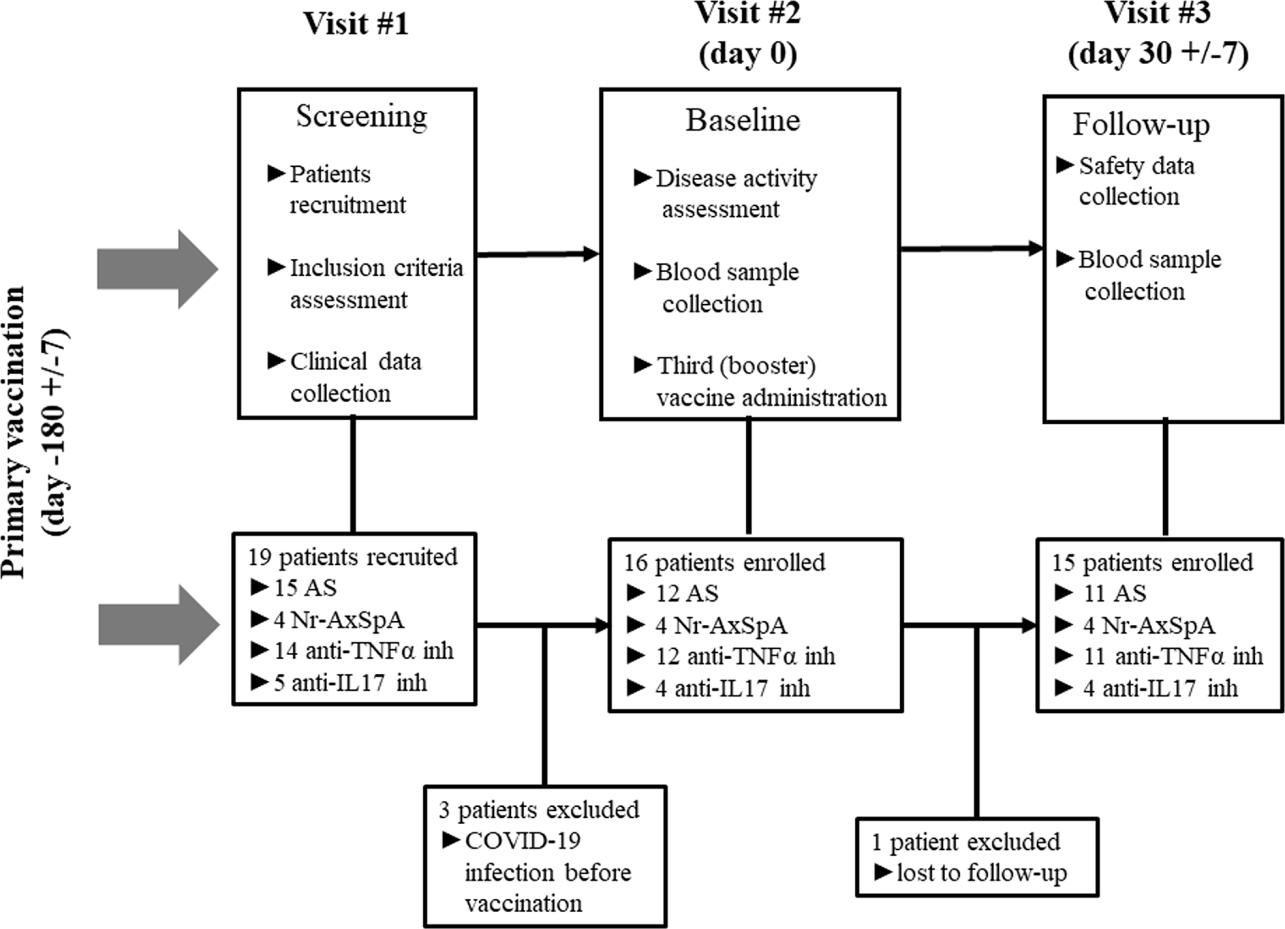
Study scheme (AS, ankylosing spondylitis; Nr-AxSpA, non-radiographic axial spondyloarthritis; TNFα, tumor necrosis factor alpha; IL, interleukin; COVID-19, coronavirus disease 19).

The study design is presented as a flowchart in **Figure 1**.

The study was conducted from March to November 2021 in accordance with the ethical standards of the Declaration of Helsinki and was approved by the Motol University Hospital Ethics Committee (EK-1729/20; issued on January 06, 2021).

### Study population

In this study, we enrolled only patients who met the following inclusion criteria (1): fulfilled the Assessment of Spondyloarthritis International Society (ASAS) classification criteria (16) (2); were receiving bDMARD therapy that was initiated at least 3 months before study enrolment (3); had completed the primary vaccination schedule (two doses of 0.3 mL/30 μg BNT162b2 vaccine with a 3-week inter-dose interval (4); had a 6-month interval following the completion of the primary vaccination schedule; and (5) provided written informed consent in accordance with the Declaration of Helsinki. Based on the “COVID-19 (SARS-CoV-2) infection” status, the participants were divided into two groups: “vaccination only” and “hybrid” (RT-PCR-confirmed SARS-CoV-2 infection after the third vaccine dose). In accordance with the guidelines, RT-PCR tests were performed in patients with symptoms of respiratory tract infection or following contact with suspected or SARS-CoV-2-positive individuals (17). The findings from these groups were compared with those of age- and sex-matched healthy controls (HC).

### Clinical and safety data collection

Clinical data (including diagnosis, treatment, age, and sex) were provided by dispensary rheumatologists. Disease activity was evaluated using the Bath Ankylosing Spondylitis Disease Activity Index (BASDAI) and Ankylosing Spondylitis Disease Activity Score (ASDAS). BASDAI ≥4 and ASDAS ≥2.1 indicate high disease activity, representing one of the eligibility criteria for initiation of the bDMARD therapy (18). Adverse events (AE) were reported using the Patient Clinical Questionnaire (PCQ) that focused on local (injection-site reactions), systemic reactions (fever, headache, myalgia, and arthralgia), and emergency medication (e.g., analgesic/antipyretic drugs). Pain intensity was assessed using a 100-point visual analog scale (VAS). Severe adverse events (SAEs) were defined as acute conditions necessitating hospitalization or urgent medical intervention that occurred after vaccination. The severity of COVID-19 in RT-PCR-positive AxSpA patients was graded as (1): asymptomatic (2); mild (infection requiring symptomatic treatment only) (3); moderate (infection requiring antiviral therapy, antivirotics, and/or anti-SARS-CoV-2 monoclonal antibodies); and (4) severe infection (requiring oxygen therapy and/or hospitalization).

### Humoral response assessment

ELISA COVID-19 receptor-binding domain (RBD) immunoglobulin G (IgG) (Test-Line Clinical Diagnostics, Brno, Czech Republic) was used for the measurement of anti-SARS-CoV-2 IgG titers (positive cutoff value >18 U/mL); the anti-nucleocapsid (NCP), anti-spike 2, and RBD antibodies (positive cutoff value >180 U/mL) were measured by immunoblot assay (IB; Microblot-Array COVID-19 IgG, Test-Line Clinical Diagnostics, Brno, Czech Republic). Responders were defined as individuals in whom anti-RBD SARS-CoV-2-specific antibodies higher than the positive cutoff level were detected by IB and ELISA after 1 month.

In addition, we tested the virus-neutralizing properties of the sera. The virus neutralization test (VNT) was performed according to a previously published protocol (19). Briefly, the SARS-CoV-2 strains used for VNT were extracted from a clinical sample (hCoV-19/Czech Republic/NRL_9640/2020|EPI_ISL_626593) and CV-1 cells (African green monkey kidney fibroblasts). Serum samples were diluted twofold and, after mixing with the virus, resulted in a final serum concentration of 1/10 to 1/2560. The results of the VNT are expressed as a virus neutralization titer, which represents an inverted value of the highest dilution of the sample that neutralizes the cytopathic effect of the virus by more than 50% (TCID50). Positivity was determined based on a titer ≥20. The response was defined as the concentration or titer of antibodies that exceeded the positive cutoff value.

### T–cell response assessment

Culture and stimulation assays were performed according to previously described methods (20). Briefly, peripheral blood mononuclear cells (PBMCs) were stimulated using PepMixTM SARS-CoV-2 S-RBD (0·1 µg/µL; JPT Peptide Technologies GmbH, Berlin, Germany) and/or 5 µL BD Fast Immune™ CD28/CD49d (BD Biosciences, San Jose, CA, USA). Moreover, stimulation with anti-human CD3-purified low endotoxin (Exbio, Vestec, Czech Republic) and the costimulatory antibody CD28/CD49d served as the positive controls.

After a total incubation of 6 hours, the stimulated PBMCs were stained with fluorescence-activated cell sorting (FACS) monoclonal antibodies against CD3 (Alexa 700; Exbio), CD4 (PE-Cy7; Exbio), and CD8 (PE-Dy594; Exbio). After fixation and permeabilization with 10× permeabilization buffer, eBioscience™ Fixation/Perm diluent, and Fixation/Permeabilization Concentrate (Thermo Fisher Scientific), the PBMCs were further stained with antibodies against Interferon gamma (IFNγ–FITC) (Exbio) and TNFα–BV421 (Biolegend) for the detection of intracellular cytokine production. Finally, samples were analyzed on a FACS Fortessa flow cytometer (BD Biosciences, San Diego, USA) and data were analyzed using FlowJo (version 10.6.1, BD Biosciences). The production of intracellular cytokines was calculated as the difference between stimulated and unstimulated (𝛥CD4+IFNγ+ or 𝛥CD4+TNFα+) cells. The T–cell response was defined as a 1.5-times increase in the proportion of CD4+IFNγ+ and/or CD4+TNFα+ cells after specific stimulation with SARS-CoV-2-derived peptides (>1.5 response rate).

### Statistical analysis

The mean and standard deviation (SD) were calculated for continuous data (age, BMI, and laboratory parameters). Statistically significant differences in the means were assessed by the Mann–Whitney *U* test for unpaired data with non-normal distribution and by the Wilcoxon test for paired data with non-normal distribution. Normality was tested using the Shapiro–Wilk normality test. The proportions were calculated for attributive data. Statistically significant differences in proportions were evaluated using Fisher’s exact test. Statistical significance was reached when *p*<0.05 in the following ranges: *p*<0.05 (*), *p*<0.01 (**), *p*<0.001 (***), and *p*<0.0001 (****). Statistical analyses were performed using GraphPad Prism 8 (GraphPad Software, San Diego, CA, USA).

## Results

### Baseline characteristics

In total, 15 participants were enrolled in the study, of whom 11 were treated with TNFα inhibitors (73·3%) and 4 with anti-IL-17 therapy (26·7%). The mean (SD) age was 43·27 ( ± 7·7) years, the disease duration was 9·53 ( ± 6·12) years, and the therapy duration was 3·87 ( ± 2·64) years. Four patients (26·7%) were diagnosed with non-radiographic AxSpA. Extraskeletal manifestations were present in seven patients (46·7%), including four cases each (26·7%) of uveitis and psoriasis. Two patients (13·3%) required comedication and dailyuse of NSAIDs or methotrexate. The majority of the patients were in remission or showed low disease activity. The mean values of ASDAS and BASDAI were 1·31 ( ± 0·45) and 1·68 ( ± 1.41) points, respectively. The mean (SD) C-reactive protein (CRP) concentration was 3·26 mg/L ( ± 2.19 SD). There were no statistically significant differences between the anti-TNFα and anti-IL-17 groups. Four patients (26.7%) were diagnosed with RT-PCR-confirmed SARS-CoV-2 infection within the first month after receiving the third BNT162b vaccine dose (booster). Patient characteristics are summarized in **Table 1**.

**Table 1:** Baseline characteristics of participants with axial spondyloarthritis (AxSpA).

	ALL (n = 15)	Anti-TNFα (n = 11)	Anti-IL-17 (n = 4)	*p*-value
Age, years, mean ( ± SD)	43·27 (7·7)	42·55 (7·44)	45·25 (9·25)	0·83
Sex, males, n (%)	15 (100)	11 (100)	4 (100)	0·99
Disease duration, years, mean ( ± SD)	9·53 (6·12)	10·64 (6·79)	6·5 (2·08)	0·47
Therapy duration, years, mean ( ± SD)	3·87 (2·64)	4·27 (2·87)	2·75 (1·71)	0·44
Nr-AxSpA, n (%)	4 (26·7)	2 (18·2)	2 (50)	0·52
Extraskeletal manifestation, n (%)	7 (46·7)	5 (45·5)	2 (50)	0·99
*Uveitis*, n (%)	(26·7)	2 (18·2)	2 (50)	0·52
*Psoriasis*, n (%)	4 (26·7)	4 (36·4)	0	0·52
Co-medication, n (%)	2 (13·3)	2 (18·2)	0	0·99
*duNSAIDs*, n (%)	1 (6·7)	1 (9·1)	0	0·99
*csDMARDs*, n (%)	1 (6·7)	1 (9·1)	0	0·99
ASDAS, points, mean ( ± SD)	1·31 (0·45)	1·21 (0·48)	1·58 (0·24)	0·14
BASDAI, points, mean ( ± SD)	1·68 (1·41)	1·52 (1·46)	2·07 (1·41)	0·54
SJC, number, mean ( ± SD)	0	0	0	0·99
TJC, number, mean ( ± SD)	0·33 (0·9)	0·45 (1·04)	0	0·79
CRP mg/L, mean ( ± SD)	3·26 (2·19)	2·56 (1·74)	5·18 (2·30)	0·08
COVID-19 (post-dose), n (%)	4 (26·7)	3 (27·3)	1 (25)	0·99

At baseline, there was no differences between the anti-TNFα and anti-IL-17 treatment cohorts.

TNFα, tumor necrosis factor-alpha; IL, interleukin; Nr-AxSpA, non-radiographic AxSpA; duNSAIDs, daily use of non-steroidal anti-inflammatory drugs; csDMARDs, conventional synthetic disease-modifying drugs; ASDAS, Ankylosing Spondylitis Disease Activity Score; BASDAI, Bath Ankylosing Spondylitis Disease Activity Index; SJC, swollen joint count; TJC, tender joint count; CRP, C-reactive protein; SD, standard deviation.

### Persistence and re-induction of humoral responses

**Figure 2:**
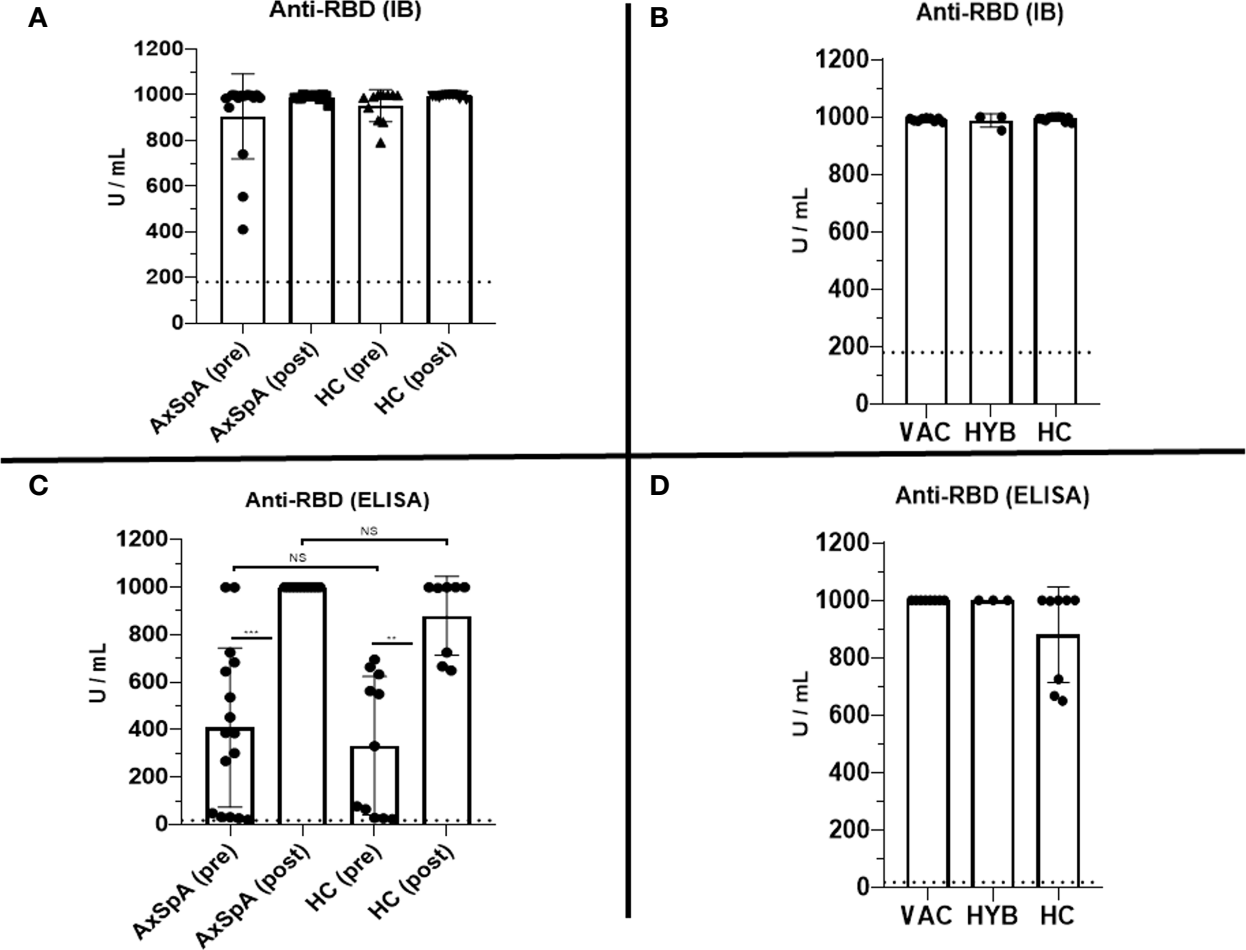
Pre-dose (6 months after completing the primary vaccination scheme) and post-dose (1 month after booster vaccination) humoral immune responses were measured as the serum concentration of anti-RBD-specific antibodies (U/mL) using Immunoblot [IB, **(A)**] and ELISA **(C)** in the cohorts of axial spondyloarthritis (AxSpA) and healthy control (HC) cohorts, “vaccinated only” (VAC) and “hybrid immunity” (HYB) AxSpA subgroups **(B, D)**; positive cutoff values (>180 U/mL for IB and >18 U/mL for ELISA) are marked as dashed line; statistically significant differences are marked as **p < 0.01, ***p < 0.001,, NS (not significant) >0.05.

**Figure 3:**
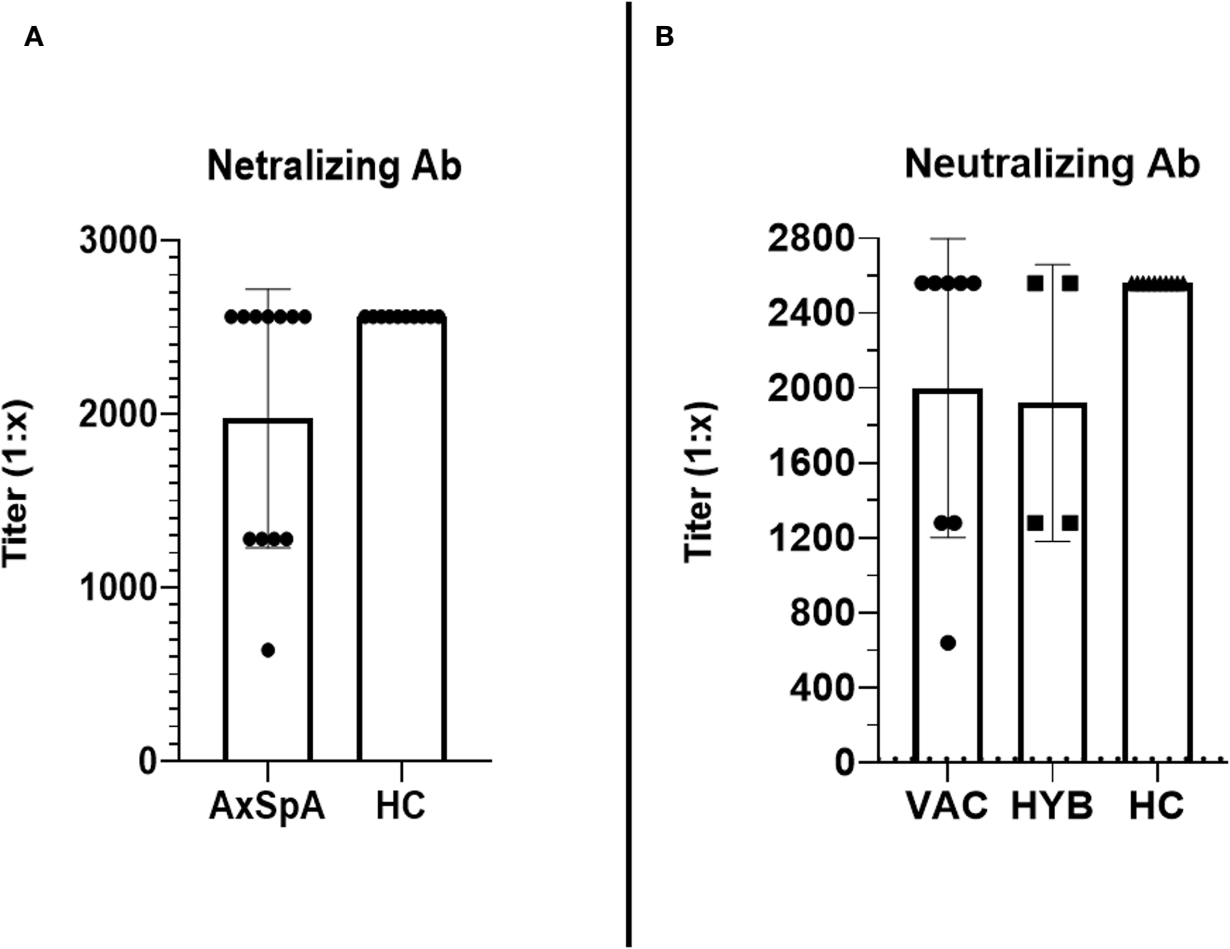
The comparison of the titers (reaching from 1:10 to 1:2560) of virus-neutralizing antibodies between Axial spondyloarthritis (AxSpA) and healthy control (HC) cohorts **(A)**, “vaccinated only” and “hybrid immunity” AxSpA subgroups **(B)**; no statistically significant differences found between cohorts.

At baseline, which represents the data 6 months after the primary vaccine schedule, the humoral response was present in all patients with AxSpA as well as in HC and was measured by IB (>180 U/mL) and ELISA (>18 U/mL). In AxSpA, the mean (SD) concentration of anti-RBD SARS-CoV-2-specific antibodies was 905·6 ( ± 186·1) U/mL using IB and 409·1 ( ± 335·7) U/mL using ELISA compared with 952·8 ( ± 69·9) and 332·7 ( ± 291·7) U/mL in HC. There were no statistically significant differences between the two cohorts. One month after vaccination, the mean antibody concentrations increased to 989·7 ( ± 12·62) and 1000 U/mL in AxSpA patients and to 993·6 ( ± 7·63) and 879·8 ( ± 166·3) U/mL in HC (**Figures 2A, C**). When evaluating the humoral response separately in distinct cohorts based on COVID-19 status, we did not observe significant differences in the humoral response between HC (mean ± SD 993·6 ± 7·63 and 879·8 ± 166·3 U/mL), vaccinated only (990·4 ± 5·52 and 1000 U/mL) and hybrid patient groups (987·9 ± 23·29 and 1000 U/mL) (**Figures 2B, D**). Similar findings were observed with anti-SARS-CoV-2 neutralizing antibodies (**Figures 3A, B**). Whereas the mean titer of virus-neutralizing antibodies was 2560 in HC, it reached 2000 ( ± 797·7) in “vaccinated only” and 1920 ( ± 739) in the “hybrid immunity” groups.

### T–cell immune response after primary and booster vaccination

**Figure 4:**
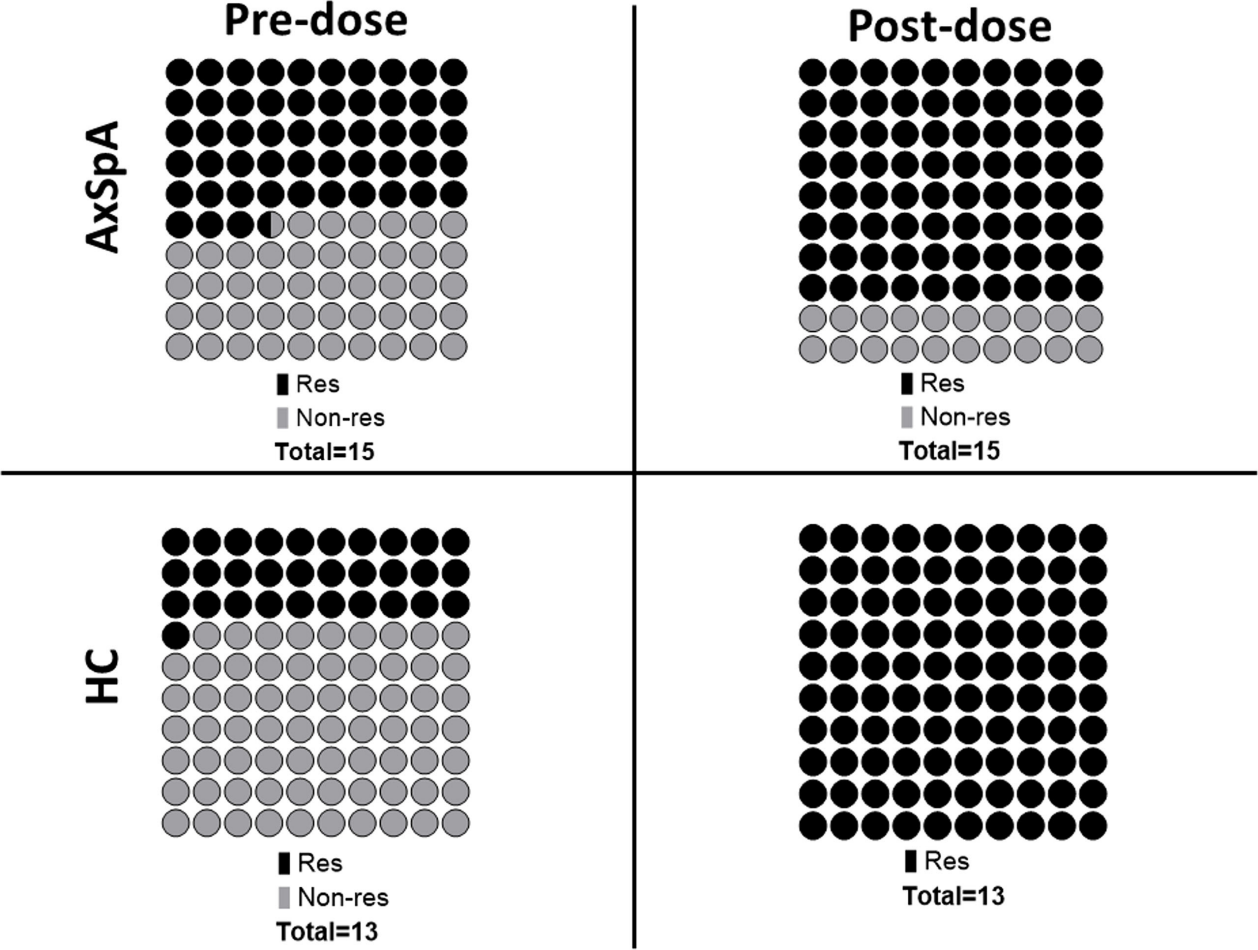
Pre- (6 months after primary vaccination scheme) and post-dose (1 month after booster vaccination) proportions of T cell immune responses (defined as 1.5-time increase in the proportion of CD4+IFNγ+ and/or CD4+TNFα+ cells after specific stimulation with SARS-CoV-2 derived peptides) between Axial spondyloarthritis (AxSpA) and Healthy control subjects (HC); responders marked as dark beads, non-responders marked as light beads, one bead represents 1% (of total); the differences between cohorts were not statistically significant.

**Figure 5:**
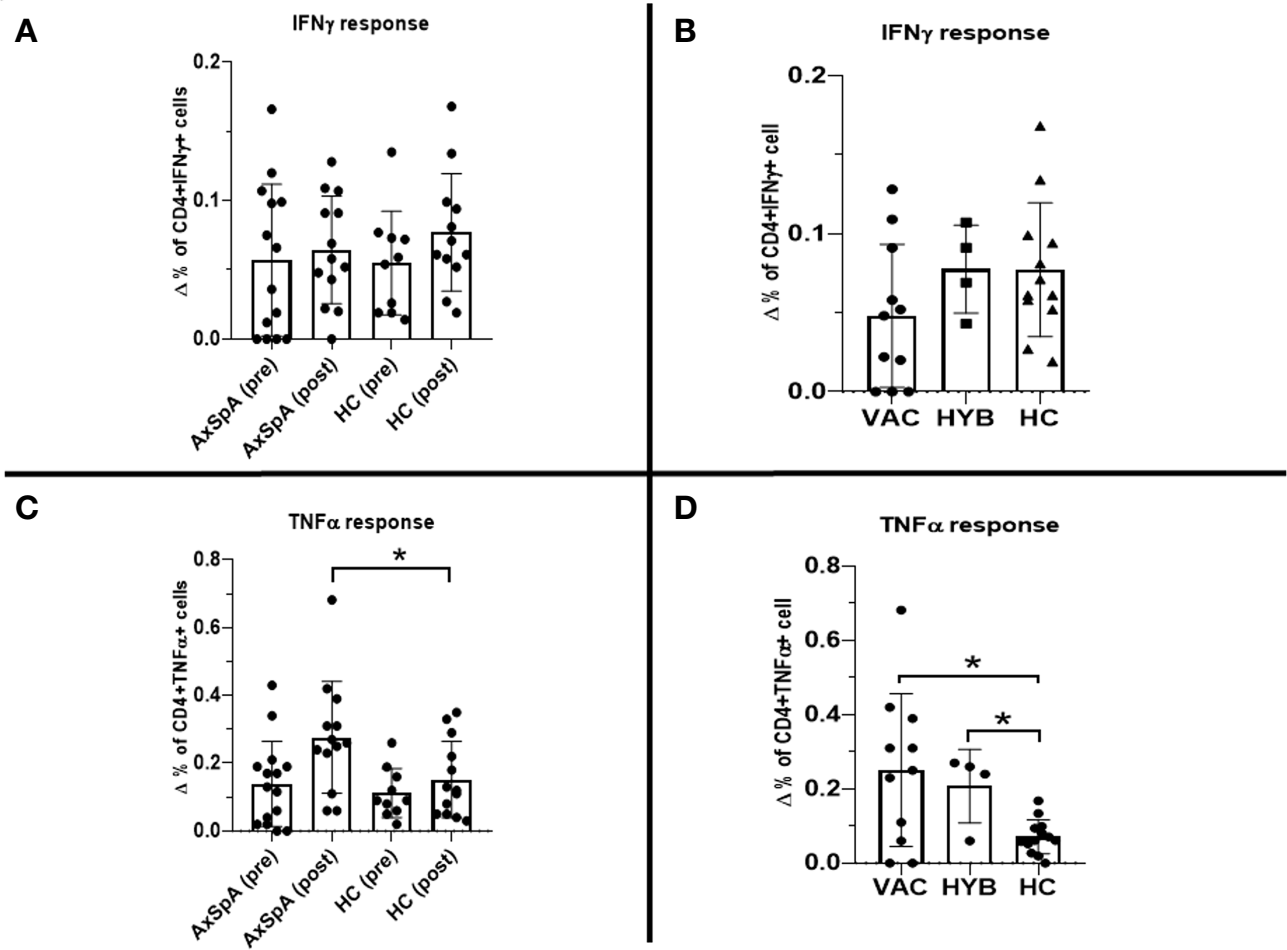
Pre-dose (6 months after primary vaccination scheme) and post-dose (1 month after booster vaccination) Interferon gamma (IFNγ) T–cell immune response measured as difference in the production of intracellular cytokine production between stimulated and unstimulated **(A)**: ΔCD4+IFNγ+; **(C)**: ΔCD4+TNFα+) cells in axial spondyloarthritis (AxSpA) patients and in healthy controls (HC), “vaccinated only” AxSpA (VAC), hybrid immunity” AxSpA (HYB) cohorts respectively **(B, D)**; statistically significant differences marked as *p < 0·05.

Six months after the second dose of BNT162b2 vaccine (primary vaccination schedule), “*in-vitro*” T–cell immune response to RBD SARS-CoV-2 peptides persisted in 30·8% (n=4/13) of HC and 53·3% (n=8/15) of AxSpA patients. One month after the administration of the third (booster) dose, the proportion of responders increased in both groups to 80% (12 out of 15 AxSpA patients) and 100% (13 HC subjects) (**Figure 4**). Nevertheless, the mean IFNγ production (Δ% of CD4+IFNγ+ cells) remained unchanged in both groups (without statistically significant changes; **Figure 5A**). The IFNγ response was unaffected by concomitant SARS-CoV-2 infection (**Figure 5B**), but we found significantly (*p*=0·04) higher TNFα responses (as Δ% of CD4+TNFα) in AxSpA patients after vaccination (0·28% ± 0·16) compared to that in HC (0·15% ± 11; **Figure 5C**). The greater response (*p*<0.05) occurred independently of the participant’s COVID-19 status (**Figure 5D**).

### Safety

**Figure 6:**
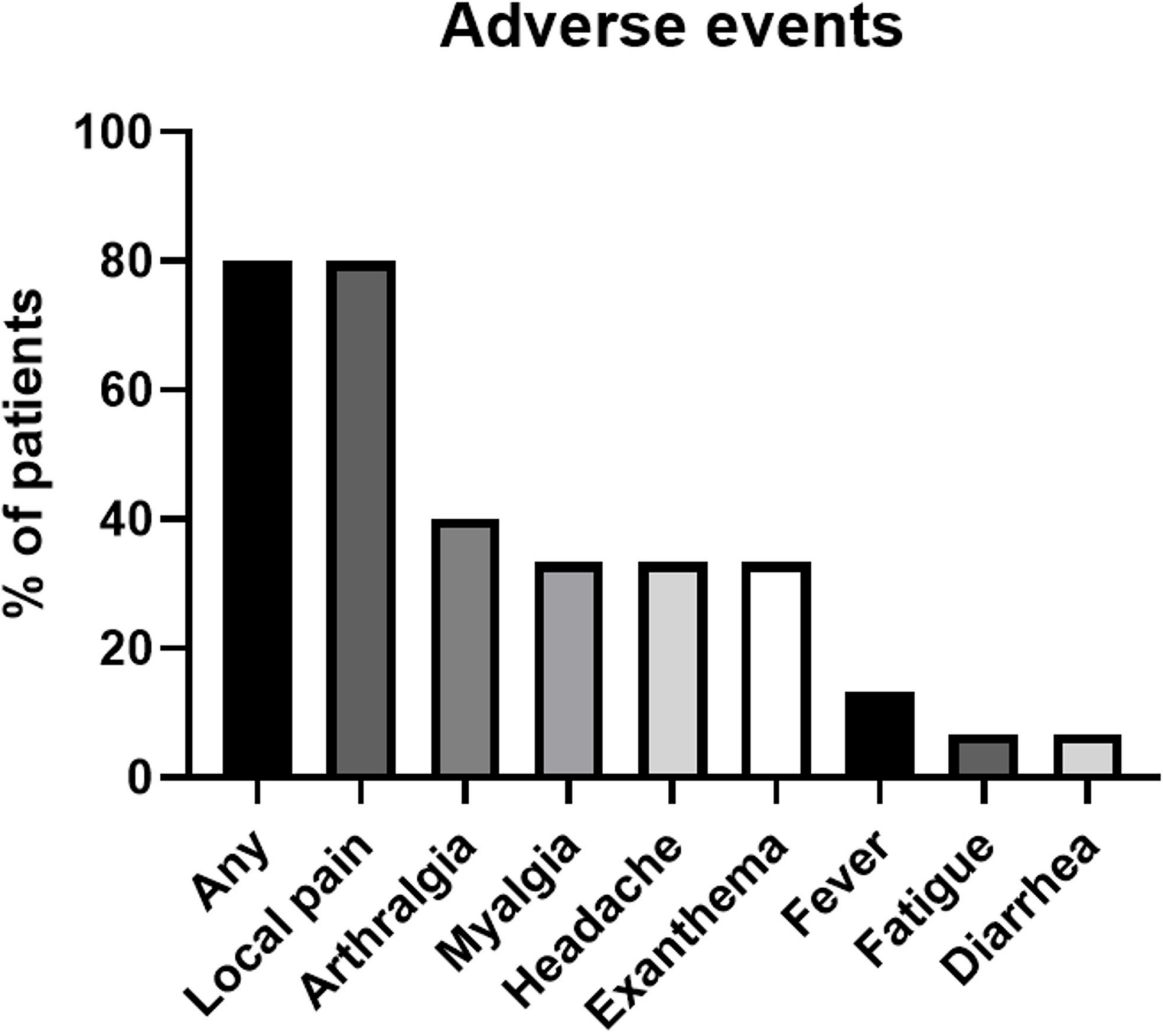
The spectrum of adverse events as a proportion of patients (%) reported by patient clinical questionnaire within the 7-day follow-up period after booster vaccination.

No SAE occurred after the administration of the third booster vaccine dose, whereas AE were reported in 80% (n=12/15) of participants within the 7-day follow-up period. The most frequent AEs were local pain at the injection site (80%, n=12/15), arthralgia (40%, n=6/15), myalgia (33·3%, n=5/12), headache (33·3%, n=5/12), and localized exanthema at the application site (33·3%, n=5/12). The spectrum of AE is summarized in **Figure 6**. The mean (SD) duration of AE was 3·3 days ( ± 2·5), and ranged from 1 to 8 days. The mean (SD) value of PGA-VAS (100) was 25·36 ( ± 24·06) and ranged from 0 to 70. Three patients required emergency medication, including NSAIDs (ibuprofen, paracetamol, and acetylsalicylic acid).

## Discussion

Anti-SARS-CoV-2, particularly mRNA vaccines, show high levels of immunogenicity and efficacy, and thereby play an important role in the struggle against the COVID-19 pandemic. Unfortunately, positive responses are limited by the waning of the immune response over time. Numerous studies have shown that the humoral response substantially decreased by 5–6 months after vaccination. The decline in response was pronounced mainly in older (age >55 to 65 years) and immunocompromised patients in whom booster vaccination should be prioritized (21, 22). Recommendations for booster vaccinations are specified in international guidelines (15). However, the immunogenicity, efficacy, and safety of booster vaccinations remain largely unclear in the majority of IMIDs.

In this study, we aimed to investigate the impact of TNFα and IL-17 inhibitors on booster vaccination with BNT162b2 in a cohort of AxSpA patients. First, we evaluated the persistence of humoral and cellular responses before booster vaccination (6 months after completing the primary schedule). We did not find any significant differences between AxSpA patients and HC, which suggests a negligible effect of biologics on response waning that was comparable to that of HCs. No decline in the total serum concentration of specific anti-SARS-CoV-2 antibodies, virus-neutralizing antibodies, or T–cell immune response was observed, whereas other studies that evaluated the immunogenicity of the anti-SARS-CoV-2 vaccine in patients with anti-TNFα inhibition reported a significant decrease in specific antibodies at the end of the 6-month follow-up period (23). Similar to our results, the T-cell immune response remained unaffected compared to that in HC (24).

Next, we investigated the immunogenicity of the third booster dose a month after vaccination. High titers of specific anti-RBD SASRS-CoV-2 antibodies and virus-neutralizing antibodies were found in all vaccinated AxSpA patients who were on bDMARDS and had enrolled in the study. Moreover, we detected T–cell immune responses in a high proportion of AxSpA patients. Both humoral and T–cell responses were comparable to those of the HC. The beneficial effects of booster vaccination have been confirmed in other studies. The booster dose was capable of inducing a response even in seronegative patients who did not respond to the standard dosage regimen, irrespective of heterologous or homologous vaccination (25, 26). Furthermore, determined the differences in immunogenicity of “vaccinated only” and “hybrid immunity”. However, we did not observe any significant differences in the humoral and T–cell immune responses. However, the “hybrid immunity” response is mainly reflected in the reduction of reinfection and poor outcomes (27) or prolonged protection against SARS-CoV-2 infection for up to 1 year (28). It is important to mention that particular studies have used different methods for humoral and cellular response assessments. Therefore, these findings must be compared and interpreted cautiously. Moreover, the level of specific anti-SARS antibodies or T–cell responses that predict immune protection remains unknown, with the exception of neutralizing antibodies (29, 30). The efficacy of vaccination must be confirmed through long-term observations. The final part of our study focused on safety. No severe AE occurred, and the AE spectrum did not differ from that of the general population (31).

## Conclusions

Despite the limitations of our study, including the small sample and the short follow-up interval after booster vaccination, our data provide important insights into the immune response persistence, immunogenicity, and safety of the third booster dose of the BTN162b vaccine in patients with AxSpA who were treated with different bDMARDs. Thus, we conclude that TNFα and IL-17 inhibitors do not impair humoral and T–cell immune responses. These conclusions support the recommendations for patients with IMIDs, which can be administered as disease-modifying antirheumatic drugs, including TNFα inhibitors, with the exception of B–cell depletion therapy (32). Neither therapy affected persistence of the immune response. Administration of the booster dose was unassociated with new safety concerns. The AE spectrum was comparable to that of the general population. Despite the increasing evidence on the efficacy and safety of the currently recommended vaccination schemes, many questions with regard to the waning immunity of the booster vaccination, which indicates the need for a second booster dose, or the efficacy against new virus variants in IMIDs or other immunocompromised patients remain unclear.

## Data availability statement

The raw data supporting the conclusions of this article will be made available by the authors, without undue reservation.

## Ethics statement

The studies involving human participants were reviewed and approved by Motol University Hospital Ethics Committee (EK-1729/20; issued on January 06, 2021). The patients/participants provided their written informed consent to participate in this study.

## Author contributions

JS contributed to the conceptualization, formal analysis, methodology, visualization, and writing of the original draft. TM contributed to conceptualization, data curation, formal analysis, funding acquisition, investigation, methodology, visualization, and writing the original draft. MR contributed to the investigation, methodology, and validation. HZ contributed to the investigation, methodology, and validation. JH contributed to the investigation, methodology, and validation. AS supervised, validated, wrote, reviewed, and edited the manuscript. RH contributed to the conceptualization, data curation, investigation, methodology, supervision, validation, writing, review, and editing. All authors contributed to the article and approved the submitted version.

## Funding

RH received funding from the Czech Health Research Council (AZV project no. NU20-05-00320) and the Ministry of Health, Czech Republic – conceptual develop- ment of research organization, Motol University Hospital, Prague, Czech Republic (00064203) TM from Technology Agency of the Czech Republic (grant no. TJ04000443) and the Czech Health Research Council (AZV project no. NU22-05-00402); and JS from the Grant Schemes at Charles University (reg. no. CZ.02.2.69/0.0/0.0/19_073/0016935). The funding agencies had no role in study design; in the collection, analysis and interpretation of data; in the writing of the report; and in the decision to submit the article for publication.

## Acknowledgments

The study was conducted in collaboration with rheumatologists from the Department of Pediatric and Adult Rheumatology, University Motol Hospital, Prague, Czech Republic who provided dispensary care for the participants, and with physicians from the Vaccination Center, University Motol Hospital, Prague, Czech Republic who administered the vaccines.

## Conflict of interest

The authors declare that the research was conducted in the absence of any commercial or financial relationships that could be construed as a potential conflict of interest.

## Publisher’s note

All claims expressed in this article are solely those of the authors and do not necessarily represent those of their affiliated organizations, or those of the publisher, the editors and the reviewers. Any product that may be evaluated in this article, or claim that may be made by its manufacturer, is not guaranteed or endorsed by the publisher.

## References

[1] Yang J Zheng Y Gou X Pu K Chen Z Guo Q. Prevalence of comorbidities and its effects in patients infected with SARS-CoV-2: A systematic review and meta-analysis. Int J Infect Dis (2020) 94:91–5. doi: 10.1016/j.ijid.2020.03.017 PMC7194638 32173574

[2] Gianfrancesco M Yazdany J Robinson PC. Epidemiology and outcomes of novel coronavirus 2019 in patients with immune-mediated inflammatory diseases. Curr Opin Rheumatol (2020) 32(5):434–40. doi: 10.1097/BOR.0000000000000725 32675715

[3] Talic S Shah S Wild H Gasevic D Maharaj A Ademi Z. Effectiveness of public health measures in reducing the incidence of covid-19, SARS-CoV-2 transmission, and covid-19 mortality: Systematic review and meta-analysis. BMJ (2021) 375:e068302. doi: 10.1136/bmj-2021-068302 34789505 PMC9423125

[4] Pak A Adegboye OA Adekunle AI Rahman KM McBryde ES Eisen DP. Economic consequences of the COVID-19 outbreak: The need for epidemic preparedness. Front Public Health (2020) 8:241. doi: 10.3389/fpubh.2020.00241 32574307 PMC7273352

[5] Moghadas SM Vilches TN Zhang K Wells CR Shoukat A Singer BH. The impact of vaccination on coronavirus disease 2019 (COVID-19) outbreaks in the united states. Clin Infect Dis (2021) 73(12):2257–64. doi: 10.1093/cid/ciab079 PMC7929033 33515252

[6] Eyre DW Taylor D Purver M Chapman D Fowler T Pouwels KB. Effect of covid-19 vaccination on transmission of alpha and delta variants. N Engl J Med (2022) 386(8):744–56. doi: 10.1056/NEJMoa2116597 PMC8757571 34986294

[7] Dagan N Barda N Kepten E Miron O Perchik S Katz MA. BNT162b2 mRNA covid-19 vaccine in a nationwide mass vaccination setting. N Engl J Med (2021) 384(15):1412–23. doi: 10.1056/NEJMoa2101765 PMC7944975 33626250

[8] Liu Q Qin C Liu M Liu J. Effectiveness and safety of SARS-CoV-2 vaccine in real-world studies: a systematic review and meta-analysis. Infect Dis Poverty (2021) 10(1):132. doi: 10.1186/s40249-021-00915-3 34776011 PMC8590867

[9] Andrews N Stowe J Kirsebom F Toffa S Sachdeva R Gower C. Effectiveness of COVID-19 booster vaccines against COVID-19-related symptoms, hospitalization and death in England. Nat Med (2022) 28(4):831–7. doi: 10.1038/s41591-022-01699-1 PMC9018410 35045566

[10] Furer V Eviatar T Zisman D Peleg H Paran D Levartovsky D. Immunogenicity and safety of the BNT162b2 mRNA COVID-19 vaccine in adult patients with autoimmune inflammatory rheumatic diseases and in the general population: a multicentre study. Ann Rheum Dis (2021) 80(10):1330–8. doi: 10.1136/annrheumdis-2021-220647 34127481

[11] Mahil SK Bechman K Raharja A Domingo-Vila C Baudry D Brown MA. Humoral and cellular immunogenicity to a second dose of COVID-19 vaccine BNT162b2 in people receiving methotrexate or targeted immunosuppression: A longitudinal cohort study. Lancet Rheumatol (2022) 4(1):e42–52. doi: 10.1016/S2665-9913(21)00333-7 PMC8577228 34778846

[12] Smetanova J Strizova Z Sediva A Milota T Horvath R. Humoral and cellular immune responses to mRNA COVID-19 vaccines in patients with axial spondyloarthritis treated with adalimumab or secukinumab. Lancet Rheumatol (2022) 4(3):e163–6. doi: 10.1016/S2665-9913(21)00393-3 PMC8691856 34957418

[13] van der Heijde D Ramiro S Landewé R Baraliakos X van den Bosch F Sepriano A. 2016 update of the ASAS-EULAR management recommendations for axial spondyloarthritis. Ann Rheum Dis (2017) 76(6):978–91. doi: 10.1136/annrheumdis-2016-210770 28087505

[14] von Elm E Altman DG Egger M Pocock SJ Gøtzsche PC Vandenbroucke JP. Strengthening the reporting of observational studies in epidemiology (STROBE) statement: Guidelines for reporting observational studies. BMJ (2007) 335(7624):806–8. doi: 10.1136/bmj.39335.541782.AD PMC2034723 17947786

[15] Landewé RBM Kroon FPB Alunno A Najm A Bijlsma JW Burmester G-RR. EULAR recommendations for the management and vaccination of people with rheumatic and musculoskeletal diseases in the context of SARS-CoV-2: The November 2021 update. Ann Rheum Dis (2022). 2:1–12. doi: 10.1136/annrheumdis-2021-222006 35197264

[16] Proft F Poddubnyy D. Ankylosing spondylitis and axial spondyloarthritis: Recent insights and impact of new classification criteria. Ther Adv Musculoskelet Dis (2018) 10(5-6):129–39. doi: 10.1177/1759720X18773726 PMC6009093 29942364

[17] Arevalo-Rodriguez I Seron P Buitrago-García D Ciapponi A Muriel A Zambrano-Achig P. Recommendations for SARS-CoV-2/COVID-19 testing: A scoping review of current guidance. BMJ Open (2021) 11(1):e043004. doi: 10.1136/bmjopen-2020-043004 PMC7789202 33408209

[18] Marona J Sepriano A Rodrigues-Manica S Pimentel-Santos F Mourão AF Gouveia N. Eligibility criteria for biologic disease-modifying antirheumatic drugs in axial spondyloarthritis: going beyond BASDAI. RMD Open (2020) 6(1):1–8. doi: 10.1136/rmdopen-2019-001145 PMC7061099 32144137

[19] Šimánek V Pecen L Krátká Z Fürst T Řezáčková H Topolčan O. Five commercial immunoassays for SARS-CoV-2 antibody determination and their comparison and correlation with the virus neutralization test. Diagnostics (Basel) (2021) 11(4):1–14. doi: 10.3390/diagnostics11040593 PMC8065578 33806216

[20] Havlin J Skotnicova A Dvorackova E Hubacek P Svorcova M Lastovicka J. Impaired humoral response to third dose of BNT162b2 mRNA COVID-19 vaccine despite detectable spike protein-specific T cells in lung transplant recipients. Transplantation (2022) 106(3):e183–4. doi: 10.1097/TP.0000000000004021 PMC8862668 34856599

[21] Levin EG Lustig Y Cohen C Fluss R Indenbaum V Amit S. Waning immune humoral response to BNT162b2 covid-19 vaccine over 6 months. N Engl J Med (2021) 385(24):e84. doi: 10.1056/NEJMoa2114583 34614326 PMC8522797

[22] Menni C May A Polidori L Louca P Wolf J Capdevila J. COVID-19 vaccine waning and effectiveness and side-effects of boosters: A prospective community study from the ZOE COVID study. Lancet Infect Dis (2022) 22:1002–10. doi: 10.2139/ssrn.3980542 PMC8993156 35405090

[23] Haberman RH Um S Axelrad JE Blank RB Uddin Z Catron S. Methotrexate and TNF inhibitors affect long-term immunogenicity to COVID-19 vaccination in patients with immune-mediated inflammatory disease. Lancet Rheumatol (2022) 4(6):e384–7. doi: 10.1016/S2665-9913(22)00069-8 PMC8975261 35403000

[24] Geisen UM Sümbül M Tran F Berner DK Reid HM Vullriede L. Humoral protection to SARS-CoV2 declines faster in patients on TNF alpha blocking therapies. RMD Open (2021) 7(3):1–3. doi: 10.1136/rmdopen-2021-002008 PMC8655347 34880128

[25] Bonelli M Mrak D Tobudic S Sieghart D Koblischke M Mandl P. Additional heterologous versus homologous booster vaccination in immunosuppressed patients without SARS-CoV-2 antibody seroconversion after primary mRNA vaccination: A randomised controlled trial. Ann Rheum Dis (2022) 81(5):687–94. doi: 10.1136/annrheumdis-2021-221558 35027397

[26] Parker EPK Desai S Marti M Nohynek H Kaslow DC Kochhar S. Response to additional COVID-19 vaccine doses in people who are immunocompromised: A rapid review. Lancet Glob Health (2022) 10(3):e326–8. doi: 10.1016/S2214-109X(21)00593-3 PMC8846615 35180408

[27] Nordström P Ballin M Nordström A. Risk of SARS-CoV-2 reinfection and COVID-19 hospitalisation in individuals with natural and hybrid immunity: A retrospective, total population cohort study in Sweden. Lancet Infect Dis (2022) 22:781–90. doi: 10.2139/ssrn.4000584 PMC8971363 35366962

[28] Hall V Foulkes S Insalata F Kirwan P Saei A Atti A. Protection against SARS-CoV-2 after covid-19 vaccination and previous infection. N Engl J Med (2022) 386(13):1207–20. doi: 10.1056/NEJMoa2118691 PMC8908850 35172051

[29] Khoury DS Cromer D Reynaldi A Schlub TE Wheatley AK Juno JA. Neutralizing antibody levels are highly predictive of immune protection from symptomatic SARS-CoV-2 infection. Nat Med (2021) 27(7):1205–11. doi: 10.1038/s41591-021-01377-8 34002089

[30] Feng S Phillips DJ White T Sayal H Aley PK Bibi S. Correlates of protection against symptomatic and asymptomatic SARS-CoV-2 infection. Nat Med (2021) 27(11):2032–40. doi: 10.1038/s41591-021-01540-1 PMC8604724 34588689

[31] Moreira ED Kitchin N Xu X Dychter SS Lockhart S Gurtman A. Safety and efficacy of a third dose of BNT162b2 covid-19 vaccine. N Engl J Med (2022) 386:1910–21. doi: 10.1056/NEJMoa2200674 PMC9006787 35320659

[32] van Assen S Agmon-Levin N Elkayam O Cervera R Doran MF Dougados M. EULAR recommendations for vaccination in adult patients with autoimmune inflammatory rheumatic diseases. Ann Rheum Dis (2011) 70(3):414–22. doi: 10.1136/ard.2010.137216 21131643

